# Measuring Gamblers’ Behaviour to Show That Negative Sounds Can Reveal the True Nature of Losses Disguised as Wins in Multiline Slot Machines

**DOI:** 10.1007/s10899-020-09976-9

**Published:** 2020-09-23

**Authors:** Molly L. Scarfe, Madison Stange, Mike J. Dixon

**Affiliations:** Department of Psychology, University of Waterloo, Waterloo, ON N2L 3G1 Canada

**Keywords:** Gambling, Negative sound, Losses disguised as wins, Slot machines, Post-reinforcement pauses

## Abstract

Losses disguised as wins (LDWs) are slot machine outcomes where players gain fewer credits than they wager. Despite being losses, slot machines celebrate LDWs with positive sounds and animations, leading gamblers to respond to them as wins. It is unknown how manipulating the sound following LDWs may influence gamblers’ behaviour. In Experiment 1, participants played two conditions on a realistic slot machine simulator: a (standard) positive sound condition (LDWs paired with positive sound, losses paired with silence), and a negative sound condition (LDWs and losses paired with negative sound). We measured participants’ behavioural responses [post-reinforcement pauses (PRPs)], win estimates, and subjective experience. In the negative sound condition, participants behaviourally responded to LDWs in a more loss-like and less win-like fashion, as measured by PRPs. Win estimates were reduced, and subjective experience was significantly impacted, but only when the negative sound condition was played second. In Experiment 2, we employed a much more subtle manipulation, pairing only LDWs with negative sound, and observed similar effects. Through these two experiments, we show that pairing LDWs with negative sound is an effective way to modify players’ responses to LDWs, causing them to respond to them more like the losses they are, rather than the wins they seem.

## Introduction

Gambling has long been a part of the Canadian landscape. For many, gambling is a harmless, enjoyable activity. However, for a small subset of the population, it may morph into a serious problem impacting diverse aspects of living (Wong 2005). In Canada, the population prevalence of problem gambling (as defined by a score of 5 or above on the Problem Gambling Severity Index [PGSI]; Ferris and Wynne 2001) is thought to be around 0.6%, and range between 0.3 and 1.2% interprovincially (R. Williams, personal communication, August 18, 2020). Among the most popular types of gambling in casinos are slot machines, with 23,750 physical machines available for play in gambling facilities in Ontario alone (OLG 2019). These machines are also extremely lucrative—over 2.6 billion dollars of the OLG’s net profit in the 2018–2019 fiscal year was generated by land-based gaming revenue, which includes both slots and table games (OLG 2019). While problem gamblers only make up a small subset of the population, the revenue derived from problem gamblers is disproportionately higher than the revenue derived from non-problem gamblers (Williams and Wood 2004), a trend that has also been reflected in slot machine revenue. One study estimated that about 62% of slots revenue in Ontario is generated from problem gamblers (Williams and Wood 2007).

Not only are slot machines associated with higher rates of problem gambling than traditional methods of gambling (Barton et al. 2017), but the period between when an individual begins to gamble regularly and when they meet criteria for a gambling disorder is significantly shorter for those that gamble on slot machines compared to other forms of gambling, suggesting that slot machines may be more addictive than other forms of gambling (Breen and Zimmerman 2002). Overall, slot machines are widely available, lucrative, and for a small subset of the population, potentially addictive.

Of the slot machines currently available for play, gamblers tend to prefer multiline slot machines which allow players to bet on multiple lines (Livingstone et al. 2008; Dixon et al. 2018). These machines incorporate themed graphics, animations, and sounds. A playing session will consist of different outcomes. When players get back more than they wager on a spin, these wins are celebrated by the machine with animations and celebratory jingles. When players spin and lose their entire spin wager, these full losses are followed by silence. Additionally, researchers have found that multiline slot machines allow for a special type of losing outcome referred to as a “loss disguised as a win” (LDW). An LDW occurs when an individual bets on multiple lines and gains back only a portion of their original bet (e.g., betting 80 cents on a spin and only winning back 65 cents). Despite being a net loss, the machine celebrates these outcomes as if they were a win by positively reinforcing the player with celebratory sounds and eye-catching animations (Dixon et al. 2010).

LDWs effectively increase the perceived reinforcement rate of slot machines by including positive visual and auditory feedback to the player on winning *and* this category of losing spins, possibly enticing continued gambling despite mounting losses (Graydon et al. 2018). In one study, high-risk, moderate-risk, and low-risk gamblers played 100 spins on a slot machine game with one of three levels of LDW outcome frequency: few, moderate, or many. Following the initial 100 spins, participants could continue to gamble for as long as they wanted. During this persistence phase, high-risk gamblers voluntarily continued to play the slot machine significantly longer in the moderate LDW condition than in the low and high LDW conditions (Graydon et al. 2018). The authors suggest that this could be the result of LDWs elevating the perceived win frequency to a “sweet spot”, such that players feel like they are winning often enough to break up long chains of losses, but not so frequently that players discover that the frequency of celebratory feedback is misleading (Graydon et al. 2018). In line with this suggestion, gambling researchers have reported that slot machine gamblers employ something called the “mini-max strategy”—betting the minimum amount of credits on the maximum amount of lines (Livingstone et al. 2008; MacLaren 2015). Importantly, this “strategy” does not actually influence the amount returned to the player (the machine’s payback percentage), but it does increase the rate of LDW presentation, and subsequently, the perceived reinforcement rate of the machine.

Despite their objective value as losses, LDWs are responded to as wins physiologically, behaviourally and subjectively. In a study examining participants’ skin conductance responses (SCRs) following wins, losses, and LDWs, the reinforcing stimuli that accompanied wins was associated with increased physiological arousal, as evidenced by higher SCRs (Dixon et al. 2010). A similar pattern of increased physiological arousal was also present following LDWs, but not for full losses where the machine goes silent (Dixon et al. 2010). LDWs also affect behavioural responses known as post-reinforcement pauses (PRPs), the amount of time between the presentation of an outcome and the initiation of the next spin (Delfabbro and Winefield 1999). In general, following wins of increasing magnitude, there are concomitant increases in the amount of time between the outcome being presented and the initiation of the following spin, as though players pause to internally celebrate the win, with longer pauses for more rewarding, larger wins (Delfabbro and Winefield 1999). In a study investigating PRPs following LDWs, participants played 250 spins on both a single line game (betting 1 cent per spin) and a multiline game (betting 20 cents per spin). In the single line game, a credit gain of 2 cents resulted in a net win of 1 credit, whereas in the multiline game, an equivalent credit gain of 2 cents resulted in an LDW (or a net loss of 18 credits). Participants’ PRPs following the LDW (net loss of 18 credits) in the multiline game were no different than the small win in the single line game, suggesting that participants react to LDWs and small wins similarly (Dixon et al. 2014a). Additionally, participants significantly overestimated the number of wins they experienced in the multiline game (where they were exposed to LDWs), but not in the single line game (where they were not exposed to LDWs; Dixon et al. 2014a).

The failure of participants to recognize LDWs as losses can be seen as a failure in categorization. In multiline play it is exceedingly difficult to categorize whether you won or lost by keeping track of where the reels stop, as wins can be formed by straight-lines, zig-zag patterns and special “scatter” symbols. In such a complex situation, players may rely on a far simpler categorization rule. In basic categorization research, when faced with a complex situation, people will typically attempt to use a single dimension to categorize exemplars—they are faster to solve categorization puzzles when they can rely on one dimension than when they have to attend to multiple dimensions (Kruschke 1992). During slots play, because most spins are full losses they are followed by silence. Wins on the other hand, are always accompanied by celebratory sounds. If players attempt to use these very salient sounds as the single-dimension that differentiates winning from losing they will (understandably) mis-categorize LDWs as wins, since the sounds accompanying these outcomes are very similar to wins.

If the audiovisual characteristics of LDWs are responsible for LDWs being miscategorized as wins, then perhaps manipulating the reinforcing positive sound that accompanies LDWs might allow gamblers to recognize that these outcomes are indeed losses. If altering the sounds following LDWs effectively “un-masks” these deceptive outcomes, then players may realize these outcomes are actually net losses. To test this possibility, Dixon et al. (2015) tested three groups of participants. One group played a slot machine simulator patterned after a standard multiline game in which celebratory sounds accompanied both wins and LDWs. For this group, gamblers significantly overestimated the number of times they thought they won, and in a post-experimental session in which they were shown a number of different outcomes (losses, wins and LDWs), when asked to indicate whether they won or lost on each spin, the majority of players claimed that LDWs were wins. A second group played the simulator in which the sounds were left on for true wins but muted for the LDWs. This manipulation only nominally reduced players’ win estimates relative to a standard game and led to only a nominal (non-significant) increase in the number of players who correctly recognized that LDWs were losses. It is possible that even without the sounds, other reinforcing stimuli, such as the brightly coloured celebratory animations that accompanied the LDWs continued to mislead players, causing them to claim that they were winning outcomes. The third group of participants received a *negative* sound following both regular losses and LDWs. Having these “raspberry” sounds following both full losses and LDWs would allow players to use sound type as a single dimension rule to correctly categorize outcomes—if they heard positive sounds they won; if they heard negative sounds they lost. Pairing losses and LDWs with these negative sounds significantly reduced win overestimations compared to the standard game and caused a significantly higher percentage of participants to correctly recognize that LDWs were indeed a type of loss.

To date, no studies have investigated if introducing negative sounds following LDWs could influence participants’ *behavioural* responses to these outcomes. As previously noted, PRPs are a non-invasive means of measuring players’ reactions to different slot machine outcomes and have been used to show that in standard games with positive sounds following LDWs, players react as though they are winning outcomes (Dixon et al. 2014a). Our goal for both Experiment 1 and Experiment 2 was to show that when LDWs were paired with negative sounds, players would react differently than when they were paired with positive sounds. Unlike in the standard game where using sound to differentiate whether they won or lost leads to a failure of categorization (mis-categorizing LDWs as wins), by applying negative sounds after both full losses and LDWs, this may allow players to correctly categorize wins and losses based on the single dimension of sound type—positive sounds mean wins, negative sounds mean losses. Here we sought to show that this easy single dimension solution would change players’ behaviour following LDWs. Specifically, we sought to show that PRPs following LDWs with positive sounds would be longer (more “win-like”) whereas the PRPs following LDWs with negative sounds would be shorter (more “loss-like”).

In addition to altering behaviour, we also sought to replicate the finding that pairing LDWs with negative sounds should lead to more high-fidelity win estimates. When LDWs are followed by negative sounds, players should give win estimates that are closer to the actual number of true wins—fewer estimated wins compared to the standard game where LDWs cause players to overestimate their wins.

In Experiment 1, the introduction of negative sounds to both full losses and LDWs meant that the vast majority of spins during game play were paired with negative sounds. In Experiment 2, we paired only LDWs with negative sounds, resulting in fewer spins being paired with a negative sound and a more subtle manipulation. For this experiment our goal was two-fold. First, we aimed to further explore the scope of the effects of negative sound on player behaviour and experience. Second, and more pragmatically, if we can show that pairing only LDWs with negative sound will still result in significant behavioural changes, such a change may be more realistic for regulators to implement. Additionally, although both losses and LDWs are monetary losses, only LDWs include misleading feedback following outcome delivery. Reversing the valence of this auditory feedback may be especially convincing as a responsible gambling tool if participants still find these games enjoyable. Consequently, we included measures of flow, positive affect, and negative affect in both studies to determine how the implementations of negative sounds impacted participants’ subjective game experiences.

## Experiment 1

### Methods

#### Participants

We recruited a sample of 73 undergraduate students (63% female) from the University of Waterloo between the ages of 19 and 34 (*M *= 20.8, *SD* = 2.25). All participants were currently enrolled in psychology courses and were recruited through an online participant pool. Members of the pool are pre-screened for a number of variables. In order to be eligible, members of the pool needed to be at least 19 years old (the minimum age to play slot machine games in Ontario), have played a slot machine at least once in the past year, and not currently be in, or seeking treatment for, problem gambling. At the time of testing, 85% of participants reported playing a slot machine between 1 and 5 times in the past year, 7% reported playing between 6 and 11 times in the past year, and 1% reported playing once a month within the past year. 7% (5) participants reported that they had not played a slot machine at all in the past 12 months, even though all participants had indicated slot machine participation on the pre-screen. Since all participants indicated that they *had* played a slot machine at least once in the last 12 months when filling out the information letter, they were deemed eligible for participation.

#### Materials

##### Problem Gambling Severity Index (PGSI)

The Problem Gambling Severity Index (PGSI; Ferris and Wynne 2001) is a 9-item measure that characterizes the severity of an individual’s gambling behaviours. Items are framed within a time period of “within the past 12 months” and are answered on a 4-point Likert scale with possible total scores ranging from 0 to 27. Response anchors range from “Never” to “Almost Always”. Total scores of 0 indicate non-problem gambling, scores of 1–2 indicate low risk gambling, scores of 3–7 indicate moderate risk gambling, and scores of 8 or more indicate high risk gambling (Currie et al. 2013). The PGSI has previously demonstrated strong internal consistency (α = .84), along with strong criterion, construct, and face validity (Ferris and Wynne 2001). We chose to use the PGSI to remain consistent with past slot machine LDW research (Dixon et al. 2014b, 2015). This measure was included only to characterize our sample.

##### Game Experiences Questionnaire (GEQ)

A shortened version of the Game Experiences Questionnaire (GEQ; Ijsselsteijn et al. 2008) including the flow, positive affect, and negative affect subscales (all of which are related to overall game enjoyment and immersion) was administered to investigate how negative sounds may influence game enjoyment. The flow and positive affect subscales are both composed of 5 items, and the negative affect subscale is composed of 4 items. All subscales are answered on a 5-point scale with response anchors ranging from “Not at all” to “Extremely”. Items for each subscale are averaged, resulting in one score per construct (Ijsselsteijn et al. 2008). Each subscale has previously demonstrated satisfactory internal consistencies, with Cronbach alphas of .87, .84, and .73, for flow, positive affect, and negative affect, respectively. The GEQ has also demonstrated face, content, and criterion validity (Ijsselsteijn et al. 2008).

##### Win Estimation Question

To assess the LDW-triggered win-overestimation effect, participants responded to the question “In each game you played 200 spins. Thinking about the first/second game you played, on how many spins did you win more than you wagered? Please provide a number between 0 and 200.” (Dixon et al. 2015).

##### Behavioural Data Collection Instruments

**Fig. 1:**
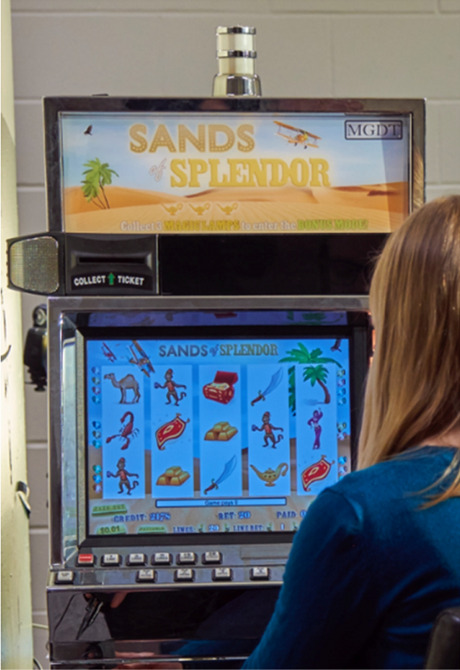
“Sands of splendor” slot machine simulator

The behavioural data were collected through a PowerLab Psychophysiology Data Acquisition System (AD Instruments), and a multiline slot machine simulation was used for game play. The slot machine simulator used in the experiment is highly realistic and presented on a computer monitor mounted inside an existing slot machine cabinet that has been used in previous studies (Graydon et al. 2018; Dixon et al. 2014a; see Fig. 1). The simulator presents time-locked markers to the PowerLab to indicate the timing of events (e.g., outcome delivery, spin initiation, etc.). A force transducer embedded underneath the spin button was used to measure the force participants apply to the spin button following each outcome (collected for reasons peripheral to the current study). LabChart 7.2 software was used to analyse the behavioural data.

#### Design

A counterbalanced within-subjects design was used such that participants either began with 200 spins on the machine that presented the positive sound condition (where a positive sound plays following both wins and LDWs), or the machine that presented the negative sound condition (where LDWs and losses are followed by a negative sound). Sound length was titrated to the amount of credits gained in each spin so that the length of each sound type (i.e., positive or negative) was identical across conditions, and the sole manipulation between conditions was the valence of the sound paired with the LDW and full loss outcomes. Outcomes were presented in one of two fixed sequences. These sequences were determined by two randomizations of the 200 spins. The order of the spins was counterbalanced such that they were reversed in the participant’s second condition (if a player was assigned to sequence 1 for their first session, they received sequence 1 in reverse order for their second session). This ensured variability in outcome order between conditions but allowed the overall number of credits gained, and the number of LDWs presented across sound conditions to be identical. The reinforcement schedules were based on the programming documents of an actual slot machine. Accordingly, full losses were the most frequently occurring outcome, followed by LDWs, small wins, and finally, large wins (see Table 1). The final counterbalanced aspect of the study was machine order, (half played the machine on the right first, half played the machine on the left first). Thus, there were 8 possible counterbalance orders (2 sounds, 2 sequences, 2 machines).

**Table 1:** Distribution of outcomes and corresponding sound pairing per condition in experiment 1

	Losses	LDWs	Small wins	Large wins
Number of spins per outcome	134	39	17	10
Sound pairing in standard condition	No sound	Positive sound	Positive sound	Positive Sound
Sound pairing in experimental condition	Negative sound	Negative sound	Positive sound	Positive sound

#### Procedure

Participants were taken to the Gambling Lab at the University of Waterloo where the experiment took place. After confirming the eligibility criteria and obtaining informed consent, participants completed the PGSI and subsequently began the experiment with either the positive sound condition or negative sound condition. The experimenter explained the basic elements of the slot machine such as how to gain credits, where the payout information was displayed, how many credits they were starting off with, the credit balance of the machine (i.e., the credit equivalent in dollars), as well as how many credits and lines they would be betting on each spin. Participants were also informed that they could win up to $10 at the end of the study depending on their final credit balance; since all spin sequences were predetermined and equated, each participant left with $10 CDN. They were then given the opportunity to ask the experimenter questions for clarification. Throughout each block, force and PRP data was recorded by the PowerLab and LabChart 7.2 software. After the first 200 spins, participants were presented with a message stating that they had reached the end of the block. Participants then filled out the shortened version of the GEQ (flow, positive affect, negative affect).

After survey completion, participants were directed to the second slot machine to complete the second session of spins. The experimenter reiterated the basic slot machine information, and once again gave participants the opportunity to ask questions. Following this, the participant proceeded to play the next slot machine game with the opposite sound valence and a reversal of spin order sequence. As with the first session, the participants completed the shortened GEQ at the end of the session. Then, participants’ win estimates for conditions 1 and 2 were gathered. Participants were asked to estimate (out of 200 spins) on how many spins they won more than they wagered. Separate estimates were gathered for each condition. At the conclusion of the study, participants were thanked for their time, given their course credit, $10 in slot machine winnings, a feedback letter, brochures on responsible gambling, and a wallet card outlining provincial mental health helplines.

#### Data Reduction

PRPs were calculated in seconds and defined as the time between when the last animated reel stopped spinning (i.e., the outcome was known), and when the participant initiated their next spin by pressing the spin button.

The different outcomes were binned into four categories: losses, LDWs, small wins (credit gains between 21 and 30), and large wins (credit gains over 30). Participants’ PRP data corresponding to each of the four bins, for each condition (positive sound/negative sound), were averaged. At the end of individual participant analyses, each participant had a total of 8 averaged data points—four outcomes (losses, LDWs small wins, large wins), for each of two conditions (positive LDW sounds, negative loss and LDW sounds) to be used in statistical analyses. Prior to calculating these averages, outliers were detected and removed using a sliding criteria for rejection based on the number of observations in the specific condition (Van Selst and Jolicoeur 1994). Trimmed means were then calculated using the outlier free averages for each outcome bin (loss, LDW, small win, large win) for each condition.

PGSI scores were calculated by summing the items. Game experience was measured by calculating the average score for each subscale (flow, positive affect, negative affect) for each condition (negative sounds, positive sounds) resulting in six data points per participant. Win estimates were calculated as the participant’s win estimates for each condition (negative sounds, positive sounds).

#### Analytical Plan

Due to the nature of the sound manipulation, we expected order effects. For example, we expected that those playing the negative sound condition first would recognize that LDWs are really losses, and this realization would influence their behavioural responses and win estimates. Such influences could manifest in that condition, but if learning takes place, could also impact estimates in the second condition. Thus, our analytical strategy was to include order as a factor in our overall analyses, and upon seeing a main effect of order or interaction involving order, we first conducted between-subjects analyses in which those who played the negative sound game first would be directly compared to those who played the positive sound game first—the only comparison uncontaminated by order effects. We then compared those who played the negative game second to those who played the positive game second. If those effects did not differ (account for the order effect or interaction), we then looked at the within subjects’ comparisons (the contrasts between positive and negative games for those playing the positive game first, and the same contrasts for those playing the negative game first). In the event of violations of sphericity, a Greenhouse–Geisser correction was applied to the degrees of freedom.

### Results

#### Problem Gambling Severity Index

Using PGSI categories established by Currie et al., (2013), 70 participants were considered low risk, and had total scores ranging from 0 to 4. Two participants fell in the moderate risk range with scores ranging from 5 to 7, and only one individual fell in the high-risk category, with a score of 8.

#### PRPs

To analyse PRP responses, a 2 (game: positive sound, negative sound) × 2 (order: positive sound first, negative sound first) × 4 (outcome: loss, LDW, small win, large win) mixed model ANOVA was conducted with order as the between-subjects variable. This analysis revealed significant main effects of game *F*(1, 71) = 5.363, *p* < .05, η_p_^2^ = .07, order *F*(1, 71) = 4.22, *p* < .05, η_p_^2^ = .056, and outcome *F*(1.05, 74.17) = 81.54, *p* < .001, η_p_^2^ = .535, along with a 3-way interaction between game, order, and outcome, *F*(3, 213) = 25.43, *p* < .001, η_p_^2^ = .264.

**Fig. 2:**
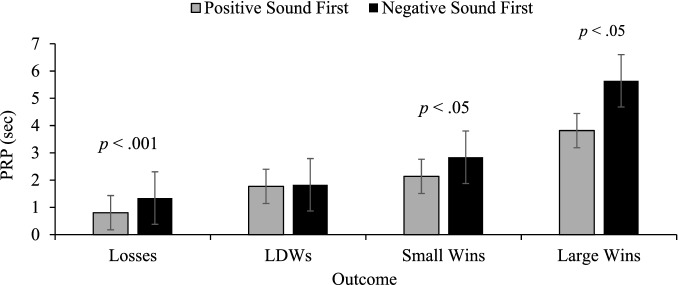
Mean PRP comparisons in game 1. Comparison of post reinforcement pauses (PRPs) for game 1, where participants played either the positive sound game first or negative sound game first. Error bars ± 1 standard error of the mean

The interaction involving order prompted the comparison of those who played the positive sounds game first to those playing the negative sound game first. This analysis revealed significant main effects of game, *F*(1, 71) = 6.52, *p* = .013, η_p_^2^ = .084, outcome, *F*(1.11, 78.59) = 105.34, *p* < .001, η_p_^2^ = .597, and a significant outcome by game interaction, *F*(3, 213) = 5.89, *p* = .001, η_p_^2^ = .077. Independent *t*-tests were subsequently conducted to uncover the source of the interaction, and it was found that the PRPs for losses, *t*(55.58) = − 5.21, *p* < .001, small wins, *t*(71) = − 2.37, *p* < .05, and large wins, *t*(71) = − 2.52, *p* < .05, but not LDWs, varied as a function of game (see Fig. 2).

We then assessed the hypotheses that positive sounds following LDWs should make them win-like, and negative sounds following LDWs should lead participants to react to them more like the losses that they are. If positive sounds make the LDWs more win-like, then there should be relatively large differences between full losses and LDWs, whereas if negative sounds make LDWs more loss-like, there should be smaller differences between full losses and LDWs. An independent *t* test on the difference scores described above showed that there were significantly larger difference scores between losses and LDWs for those playing the positive sound game than for those playing the negative sound game, *t*(48.33) = 3.62, *p* = .001. We then conducted a similar analysis, but this time comparing the differences between LDWs and small wins for those playing the positive and negative sound game. Consistent with the idea that positive sounds make LDWs more similar to wins, the differences between LDWs and small wins in the positive sound game were significantly smaller than in negative sound game, *t*(66.19) = − 3.69, *p* < .001.

A comparison of the participants who played the positive game second to those who played the negative game second indicated only a significant main effect of outcome, *F*(1.045, 74.18) = 47.71, *p *< .001, η_p_^2^ = .402. All other effects were non-significant (all *p*’s > .05).

#### Game Experiences Questionnaire and Win Estimates

Three components of the GEQ were assessed: flow, positive affect, and negative affect. In addition, we also assessed participants’ win estimates. First, to assess the impact of order on participants’ ratings, we conducted a 2 (game) × 2 (order) mixed model ANOVA with order as the between-subjects variable for each subscale, and for the win estimates. Then, if a main effect or interaction involving order was found, we followed up with independent samples *t*-tests for game 1 and game 2, comparing those that played the positive sounds with those that played the negative sounds for each game. In the case of violations of equality of variances, the degrees of freedom were corrected and reported below.

Table 2 shows the data for all three GEQ variables and the win estimates. The two counterbalanced orders are presented sequentially with the order in which they played the games. To preface the results, for those who played the game with positive sounds first (Order 1 in Table 2), there were significant differences in flow, positive, negative affect and win estimates between the positive game and the negative game. For those playing the negative game first (Order 2) there were no such differences.

**Table 2:** Subjective variable means and standard deviations for each counterbalanced order

Variable	Counterbalanced order 1		Counterbalanced order 2	
	Positive game	Negative game	Negative game	Positive game
Flow	2.49 (.89)	2.22 (.84)*	2.53 (.67)	2.30 (1.04) n.s.
Positive affect	2.53 (.90)	1.90 (.64)**	2.74 (.78)	2.78 (1.02) n.s.
Negative affect	2.53 (.83)	3.08 (.82)**	2.39 (.74)	2.53 (.96) n.s.
Win estimates	49.97 (33.17)	38.19 (32.08)**	45.65 (27.31)	44.24 (30.62) n.s.

Means (standard deviations) for flow, positive affect negative affect and win estimates for positive and negative games and the order in which games were played

*p < .05; **p < .01

##### Flow

The mixed model ANOVA revealed no significant main effects, but a significant game by order interaction, *F*(1, 71) = 8.27, *p* < .01 η_p_^2^ = .104. The independent samples *t*-tests contrasting those playing the positive sound game versus the negative sound in game 1 revealed no significant differences between groups, *t*(65.13) = -.19, *p *> .05. The game 2 comparison also did not reveal any significant differences between groups, *t*(71) = .34, *p* > .05. The interaction of game by order was caused by those playing the positive sound game first (order 1 in Table 2) experiencing significantly greater flow than the negative game (which they played second), *t*(35) = 2.46, *p* = .019. For those playing the negative game first, (order 2 in Table 2) flow did not differ between the positive sound and negative sound games, *t*(36) = − 1,71, *p* = .10.

##### Positive Affect

The mixed model ANOVA revealed a main effect of game, order, and a significant game by order interaction, *F*(1, 71) = 8.34, *p* < .01 η_p_^2^ = .105. The independent samples t-test for game 1 revealed no significant difference between groups, *t*(71) = − 1.08, *p *> .05. However, when a similar analysis was conducted for game 2, it was revealed that PA significantly decreased in the negative sound condition, *t*(60.98) = 4.45, *p* < .001. For the dependent comparisons, there were significant differences between the positive game and the negative game for order 1 in Table 2 (those playing the positive game first), *t*(35) = 4.29, *p* < .001, but not for order 2 (those playing the negative game first), *t*(36) = .309, *p* = .759.

##### Negative Affect

The mixed model ANOVA revealed main effects of game, order, and a significant game by order interaction, *F*(1, 71) = 13.32, *p* < .001 η_p_^2^ = .158. The independent samples t-test for game 1 revealed no significant difference between groups, *t*(71) = .81, *p *> .05. However, when a similar analysis was conducted for game 2, it was revealed that negative affect significantly increased in the negative sound condition, *t*(71) = − 2.63, *p* = .01. For the dependent comparisons, there were significant differences between the positive game and the negative game for order 1 in Table 2 (those playing the positive game first), *t*(35) = − 3.93, *p* < .001, but not for order 2 (those playing the negative game first), *t*(36) = 1.14, *p* = .262.

##### Win Estimates

**Fig. 3:**
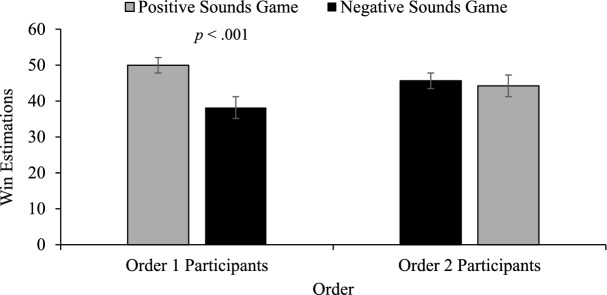
Win estimations. Win estimations across the counterbalanced orders. Order 1 participants played the positive game first, Order 2 participants played the negative sound game first. Error bars ± 1 standard error of the mean

The mixed-model ANOVA revealed a significant game by order interaction, *F*(1, 71) = 5.86, *p* < .05 η_p_^2^ = .076. No significant differences occurred for the between subjects’ comparisons (positive vs. negative game 1 or positive vs. negative game 2). The within subjects’ comparisons revealed that for those who played the positive game first, their win estimates were significantly higher for the positive game than the negative game which they played second, *t*(35) = 5.16, *p* < .001. For those playing the negative game first, their win estimates for this negative game were no different from their win estimates for the positive game which they played second, *t*(36) = .287, *p* > . 05 (see Fig. 3).

### Discussion

The results of Experiment 1 indicate that pairing both losses and LDWs with negative sounds had a significant effect on players’ behaviour and subjective experience. In terms of PRPs, when participants who played the game with positive sounds first were compared to those who played the negative game first (the only comparison uncontaminated by order) the group playing the game with positive sounds had smaller PRPs than the group playing the game with negative sounds for every outcome *except* LDWs. The most reasonable interpretation of this pattern of data is that it was due to a mixture of between group differences due to sampling error, and the effect of the positive sounds following LDWs in the (standard) positive sound condition. These between-subject differences can be seen by comparing the smaller magnitude of the white bars (those playing the game with positive sounds first), compared to the black bars (those playing the negative sounds first) for three of the four outcomes. The only outcome where the PRPs were equated was the LDWs. We interpret this effect as attributable to the positive sounds following LDWs *elevating* PRPs (players react to them as though they are wins). This elevation was not present in the group playing the negative sound game first.

Further evidence for the positive sounds elevating PRPs comes from the difference score analyses. In the positive sound game where positive sounds followed LDWs, the PRPs became less loss-like (larger differences between regular losses and LDWs) than in the negative sound game where the negative sounds made LDWs more loss-like (smaller differences between regular losses and LDWs). The difference score analyses also demonstrate that positive sounds following LDWs make players respond to them in a more win-like manner: there were smaller differences between LDWs and small wins for those playing the positive sound game, compared to the negative sound game. The negative sounds made LDWs seem less win-like, leading to larger differences between PRPs for LDWs and true wins.

We note that these difference score analyses are not subject to the complications involved in having between group differences due to sampling error. Regardless of the particular group’s overall propensity to have large or small PRPs, the positive sounds following LDWs should make them less loss-like, and more win-like. Conversely, the negative sounds following LDWs should make PRPs more loss-like and less win-like. In assessing the more subtle effects that we expect in Experiment 2, where in the negative sound condition only the LDWs will be paired with negative sounds, we will use these difference scores as a priori contrasts.

For flow, positive affect, and negative affect, there were order effects—the effects on these variables depended on which game was played first. Nevertheless, there were clear effects of the negative sounds. In terms of flow, those playing the positive sound game first endorsed deeper flow for this game than the negative game which they played after. For those playing the negative game first, there was no difference in flow ratings between the positive and negative games. A similar pattern of results emerged for both positive and negative affect. The ratings of players were only significantly different for those who played the game with positive sounds first followed by the game with negative sounds. We suggest that these relatively low frequency gamblers are taken in by the disguise of LDWs in the positive sound game when they play it first. When immediately afterward, they are exposed to a game where they can use a single dimension rule to tell whether they won or lost, these negative sounds expose LDWs for the losses that they are. This idea is supported by players’ win estimates, which are significantly lowered (e.g., more accurate) in the game with negative sounds. Taken together, the data converge to indicate that pairing negative sounds with both full losses and LDWs served to unmask the disguise in LDWs. It is important to note that it remains unclear why there were no effects for those who played the negative game first. Prior to attempting to interpret this finding, we sought to assess the reliability of this finding by replicating it in Experiment 2.

In terms of PRPs, having the negative sounds accompany LDWs made players react to them in a more loss-like fashion. The negative sound game was also associated with lowering flow compared to the positive sound game, for those who played the positive game first. In terms of harm reduction, our negative sound manipulation may provide players a more veridical representation of how often they won during play. The reduction in flow during the negative sound game could also mean that players would be less likely to spend more time and money than intended. Both of these aspects may make the inclusion of negative sounds following losses and LDWs a strategy to help counteract the harms associated with LDWs.

Unfortunately, regulators, operators, and game designers may be unlikely to adopt these measures as the negative sounds also made game play a less enjoyable experience. In this experiment, pairing both full losses and LDWs with negative sounds afforded players the ability to use a single dimension rule to tell whether they won or lost (using the positive and negative sounds, respectively). Since full losses are by far the most frequent outcome, this meant that players heard negative sounds on 86.5% of trials. Such a high percentage of “negative” spins might have led to the lowered positive affect and heightened negative affect scores observed, and would pose a relatively unpalatable harm reduction strategy for those who seek to promote the enjoyment of slots play for recreational gamblers. Therefore, in Experiment 2, we sought to further investigate the impact that negative sounds have on participant responses to LDWs, and to assess whether we could still show that negative sounds could alter the manner in which players code LDWs (reacting to them as being more loss-like in terms of PRPs and lowering win overestimations), but with a more subtle manipulation of only including negative sounds following LDWs.

## Experiment 2

### Methods

#### Participants

We recruited a sample of 70 undergraduate students (58.6% female) from the University of Waterloo between the ages of 19 and 25 (*M *= 20.8, *SD* = 1.48). Participants were screened to ensure they met inclusion criteria (described in Experiment 1). Of the 70 participants recruited, 75.7% reported playing a slot machine between 1 and 5 times in the past year, 15.7% reported playing between 6 and 11 times in the past year, and 2.8% reported playing either once a month or 2–3 times a month within the past year. 5.7% (4) of participants reported having not bet or spent money on slot machines in the past 12 months. Since all participants indicated slot machine experience at the level of the pre-screen and all participants actively endorsed this eligibility criteria during the informed consent process at the time of testing, all participants were retained for data analysis.

### Materials

All materials were identical to those described in Experiment 1.

#### Procedure

All procedures were identical to Experiment 1, with the sole exception being that *only* LDWs were paired with the negative sound (losses were followed by silence).

### Results

#### Problem Gambling Severity Index

Using established cut-off criteria (Currie et al. 2013), 68 participants were considered low risk, and had total scores ranging from 0 to 4. Two participants were in the moderate risk range with scores of 5.

#### PRPs

**Fig. 4:**
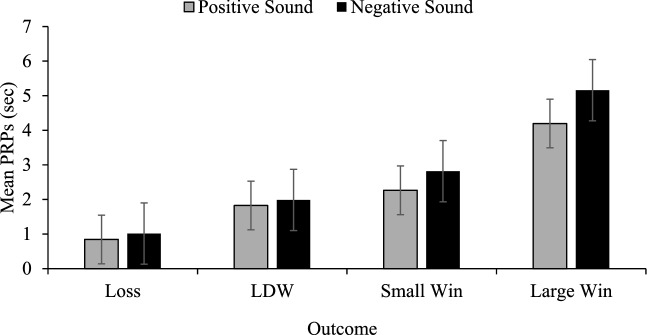
Mean PRP comparisons in game 1. Game 1 post reinforcement pause (PRP) comparisons between those that played the positive sound game first to those that played the negative sound game first. Error bars ± 1 standard error of the mean

**Fig. 5:**
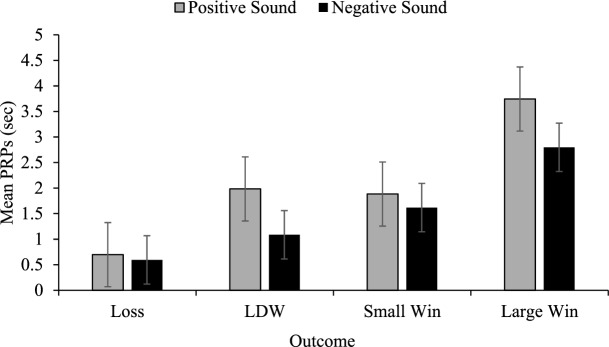
Mean PRP comparisons in game 2. Game 2 PRP comparisons comparing those that played the positive sound second to those that played the negative sound game second. Error bars ± 1 standard error of the mean

The PRP data was analyzed using a repeated measures ANOVA with game (positive sounds, negative sounds), order (positive game first, negative game first) and outcome (losses, LDWs, small wins, large wins), as factors, to examine higher order interactions. This analysis revealed a main effect of outcome, *F*(1.04, 63.66) = 72.817, *p *< .001 η_p_^2^ = .544, as well as a game by outcome by order interaction, *F*(1.25, 76.27) = 21.12, *p *< .001 η_p_^2^ = .257. As in Experiment 1, the file was split to directly compare those who played the positive game first versus those who played the negative game first, and those who played the positive game second versus those who played the negative game second (see Figs. 4, 5).

For the first game comparison, there was a main effect of outcome, *F*(1.076, 71.03) = 103.83 *p *< .001, η_p_^2^ = .611. There was no outcome by sound interaction (*p *= .216). Due to their theoretical significance, we conducted the same difference score analysis as in Experiment 1 to assess whether the positive sounds following LDWs made them more win-like compared to negative sounds, which should make LDWs more loss-like. There were no differences between the positive and negative sound games when comparing the difference scores between PRPs for losses and LDWs, *t*(66) = .089, *p *= .930. There were significant differences between the positive and negative sound games when comparing the PRP differences between small wins and LDWs, *t*(66) = 2.50, *p *= .015. As in Experiment 1, the positive sounds following LDWs made players react to them in a more win-like fashion, whereas the negative sounds created more of a distinction between these outcomes for players.

For the second game comparison, there was a main effect outcome, *F*(1.076, 71.03) = 38.51, *p *< .001, η_p_^2^ = .368, but no outcome by sound interaction (*p *= .218). There were however differences between the positive and negative sound games in terms of the difference scores between the PRPs for losses and LDWs, *t*(66) = 5.44, *p *< .001. As in Experiment 1, the positive sounds following LDWs made them less loss-like (greater differences between full losses and LDWs), whereas the negative sounds made them more loss-like (smaller differences between full losses and LDWs). There were significant differences between the positive and negative sound games when comparing the differences between LDWs and small wins, *t*(66) = 3.30, *p *= .002. Here, the positive sounds following LDWs made players react to them in equivalent manner as wins, whereas the negative sounds led to larger differences between LDWs and small wins (rendering them less win-like).

#### Game Experiences Questionnaire and Win Estimates

For all three subjective variables, there were interactions with order. The means for flow, positive and negative affect are presented in Table 3 which shows the order that the games were played in for the two counterbalanced orders. To preface the results, we observed the same pattern of order effects as in Experiment 1—there were strong significant effects of flow, positive affect and negative affect for those that played the game with positive LDWs first—playing the negative game immediately after reduced both flow and positive affect, and increased negative affect. For those playing the negative game first, playing the positive game immediately after had negligible, non-significant effects on flow, positive affect and negative affect.

**Table 3:** Subjective variable means and standard deviations for each counterbalanced order

Variable	Counterbalanced order 1		Counterbalanced order 2	
	Positive game	Negative game	Negative game	Positive game
Flow	2.48 (.84)	2.15 (.93)**	2.35 (.79)	2.37 (.98) n.s.
Positive affect	2.8 (.93)	2.19 (.87)**	2.85 (.87)	2.82 (1.12) n.s.
Negative affect	2.33 (.79)	2.64 (.81)**	2.34 (.77)	2.36 (.91) n.s.
Win estimates	49.40 (30.5)	38.49 (27.0)*	49.86 (37.35)	52.94 (48.33) n.s.

Means (standard deviations) for flow, positive affect negative affect and win estimates for positive and negative games and the order in which games were played

*p < .05; **p < .01

##### Flow

For flow, there was a main effect of game, *F*(1,68) = 5.29, *p *= .025, η_p_^2^ = .072, and a significant game by order interaction, *F*(1,68) = 4.29, *p *= .042, η_p_^2^ = .059. This interaction was caused by a significant reduction in flow among those who played the positive game first followed by the negative game second, *t*(34) = 2.86, *p *= .007. For the group that played the negative game first there was virtually no change in flow across games, *t*(34) = .178, *p *= .86.

##### Positive Affect

For positive affect, there was a significant main effect of game, *F*(1,68) = 11.85, *p *= .001, η_p_^2^ = .148, and a game by order interaction, *F*(1,68) = 13.77, *p *< .001, η_p_^2^ = .168. Those playing the positive game first experienced a significant drop in positive affect when they then played the negative game, *t*(34) = 5.51, *p *< .001. For those playing the negative game first, there was no change in positive affect when they then played the positive game, *t*(34) = .176, *p *= .86.

##### Negative Affect

For negative affect, there was a game by order interaction *F*(1, 68) = 4.95, *p *= .029, η_p_^2^ = .068. For those who played the positive game first, there was a significant increase in negative affect while playing the negative sound game second, *t*(34) = 3.03, *p *= .005. For those who played the negative sound game first there was no change in negative affect from this game to the positive game they played second, *t*(34) = .200, *p *= .843.

##### Win Estimates

**Fig. 6:**
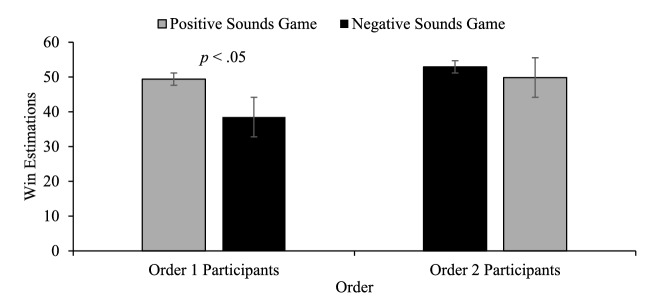
Win estimations. Win estimations across the counterbalanced orders. Order 1 participants played the positive game first, Order 2 participants played the negative sound game first. Error bars ± 1 standard error of the mean

For win estimates, there was a significant main effect of game, *F*(1, 68) = 4.41, *p *= .039, η_p_^2^ = .061, with win estimates being 7 wins lower in the negative sound condition. There was no effect of order or game by order interaction (smallest *p* value = .244). For comparison with Experiment 1, we compared the contrasts between those playing the positive game first versus negative game first. This comparison revealed no differences in win estimations. We also conducted the within-subject comparisons to see if we would replicate the findings of Experiment 1: win estimation effects only for those who played the positive game first. As in Experiment 1, there were significant differences between the positive game and negative game win estimates for those who played the positive game first, *t*(34) = 2.42, *p *= .021, but again no difference between win estimates for those playing the negative game first (see Fig. 6).

### Discussion

In Experiment 2, we were able to meet our first objective of replicating the PRP difference score findings of Experiment 1, such that negative sounds made LDW PRPs more loss-like and less win-like. In Experiment 2, the game 1 difference score analyses suggested that negative sounds following LDWs resulted in PRPs that are less win-like. The differences between LDWs and small win PRPs in the negative sound condition were significantly larger than in the positive sound condition. The game 2 difference score analyses provided support for the idea that negative sounds following LDWs results in PRPs that are both more loss-like and less win-like. Difference scores between losses and LDWs were significantly smaller in the negative sound game than they were in the positive sound game (e.g., LDW PRPs were more loss-like in the negative sound game). PRP difference scores between small wins and LDWs were significantly larger in the negative sound game than in the positive sound game (e.g., LDW PRPs were less win-like in the negative sound game).

Subjective results and win estimates in Experiment 2 replicated those of Experiment 1. In terms of participants’ subjective experience of game play, for participants who played the positive sounds game first, we found decreased ratings of flow and positive affect, and increased ratings of negative affect, in the negative sound condition when this condition was experienced second. For those that played the negative sounds game first, we found that ratings of flow, positive affect, and negative affect did not change significantly between conditions. A similar pattern was observed for win estimates. Those that played the positive sound game first had significantly decreased win estimates in the negative sound condition. Those that played the negative sound game first had win estimates that did not differ between conditions.

## General Discussion

The overarching goals of Experiment 1 and 2 were to investigate whether adding a negative sound following LDW outcomes would influence how participants behaviourally respond to these outcomes (as measured by PRPs) and to replicate past findings by Dixon et al. (2015) that negative sound increases the fidelity of participant win estimations. In Experiment 1, we paired both LDWs and losses with negative sound, which allowed participants to use this single feature as a cue to whether they had encountered a win or a loss. In Experiment 2, we sought to further explore the impact of pairing LDWs with negative sound, and further demonstrate the impact that sound may have on the misrepresentation of LDW outcomes. To do this, we used a more subtle manipulation—adding negative sound to only LDW outcomes—to investigate whether this manipulation would result in meaningful behavioural changes and higher fidelity win estimations. In both experiments, we also sought to explore how negative sound may influence participants’ subjective game experience, as measured by flow, positive affect, and negative affect. Together, Experiment 1 and 2 demonstrate novel evidence that pairing negative sounds with LDWs does indeed lead to a change in participant behaviour, such that participants’ PRPs are rendered more loss-like and less win-like. Both studies replicate past findings (Dixon et al. 2015) and provide evidence that negative sound results in higher fidelity win estimations, even when only LDWs are paired with a negative sound, as was done in Experiment 2. Finally, our set of studies demonstrates that the negative sound condition, when played second, does indeed impact the players subjective experience of the game, such that reports of flow and positive affect are decreased, and negative affect is increased.

The clearest evidence for negative sound changing the way LDWs are encoded by participants comes from changes in behavioural responses to these outcomes, as measured by PRPs. Previous research has demonstrated that as win size increases, PRP length increases concomitantly (Delfabbro and Winefield 1999). This tight titration between PRP length and win size is thought to reflect an internal celebration of the win that the participant experiences (Delfabbro and Winefield 1999). Previous research has shown that PRPs following LDWs in standard machines are the same length as those of small wins, and significantly longer than those of losses (Dixon et al. 2014a). This suggests that participants celebrate these losing outcomes and provides evidence of the deceptive nature of LDWs; participants are internally *celebrating* an outcome where they are losing money. While past studies have demonstrated the similarity in PRPs following LDWs and small wins, no studies have investigated whether this measure of behavioural reactivity changes when LDWs are paired with a negative sound. In Experiment 1, where LDWs and losses were paired with a negative sound, we found that while those that played the positive sound game first had shorter PRPs overall, by pairing LDWs with positive sound, these participants elevated their PRPs for LDWs to that of the participants that had the negative sound first, who had longer PRPs overall. This demonstrates that sound influences the way in which participants behaviourally respond to LDW outcomes.

The most compelling evidence for behavioural change across both experiments were the PRP difference score analyses. In Experiment 1, when comparing game 1 PRP difference scores between losses and LDWs for participants that played the positive game first versus those that played the negative game first, there was a significantly smaller difference between loss and LDW PRPs in the negative sound game, and a significantly larger difference between loss and LDW PRPs for those that played the positive game. This suggests that negative sound makes LDWs more loss-like. In Experiment 1, we were also able to show that negative sounds made LDWs less win-like. Participants’ difference scores between small wins and LDWs revealed that when LDWs are paired with a negative sound, there is a significantly larger difference between LDW and small win PRPs than when they are paired with positive sound. In this study, participants were able to use sound as a way to categorize whether an outcome was a win or loss by attending to the sound played following the spin. These PRP results show that negative sound made LDWs both more loss-like, and less win-like. Together, this provides support for the idea that participants successfully used sound as a way to categorize whether they had won or lost.

The PRP effects in the difference score analyses were also present in Experiment 2, where only LDWs were paired with the negative sound. While we did not find evidence for negative sound making LDWs more loss-like in game 1 comparisons, we replicated the finding that negative sounds made LDWs less win-like. That is, the PRP differences between small wins and LDWs were significantly more pronounced in the negative sound group compared to the positive sound group. For game 2 comparisons, we found evidence for both statements—negative sound made participants respond to LDWs both in a more loss-like, and less win-like fashion. Together, Experiment 1 and 2 show that manipulating the sound paired with LDWs effectively alters participants’ PRPs to be more loss-like and less win-like following these outcomes. This finding contributes to a growing body of LDW research, and provides evidence that the use of negative sound in slot machine games impacts behavioural responses to LDWs.

Negative sounds were also shown to be an effective way to reduce participants win over-estimates. In both experiments, participants had higher fidelity win estimations in the negative sound condition. Interestingly, this win estimation decrease was only present for the group that played the positive game first. For those that played the negative sound game first, win estimates did not differ between conditions. It is possible that our sampling of undergraduate students with somewhat limited slot machine gambling experience may have led to this unexpected finding. A more experienced sample of multiline slot machine gamblers might expect a high rate of positive reinforcement to be emitted by the machine, such as in the positive sound condition. For an experienced group of gamblers, a stark contrast would emerge between their expectancies of multiline games gleaned from their playing experience, and their experience of playing the game presented in the negative sound condition, even if it were played first. They would realize that there are far fewer spins accompanied by positive sounds than they are used to—a situation that would likely impact win estimates, regardless of whether it came first or second. In contrast, a sample of less frequent slots players, as in the present sample, would not have a well-formed template of what to expect in terms of celebratory sound frequency when playing a multiline slots game. These participants would not find the lower-than-normal celebratory sound frequency associated with the negative sound game jarring if they had limited slots experience. In fact, they may use the negative sound game as a template for what should be expected in terms of celebratory sound frequency. Participants playing the negative sound game first would however be exposed to an incongruency between sound and visual animations on LDWs. They would see “dancing” celebratory animations but hear negative sounds—rendering the disguise associated with LDWs ineffective. When they play the positive game second, they realize that there is a discrepancy between the template they have recently formed, and the much higher rate of celebratory sounds—and this discrepancy may cause suspicion, and a closer look at the various outcomes, including noting that the amount “won” on LDWs is less than their total bet. Thus, the disguise in losses disguised as wins would not be formed in either game 1 or game 2 leading to no differences between the negative and positive games.

By contrast, those who played the positive game first would use this high-frequency of celebratory sounds as the template of what to expect while playing a multi-line game. For these players, there is no incongruency on LDWs—they see positive visuals, and they hear positive sounds—a combination that would disguise the fact that they were losing money on those spins. When playing the negative sound game second, there would be far fewer celebratory sounds overall and negative sounds indicating that LDWs were losing outcomes. Furthermore, there would be an incongruency between the valence of the sound and animations accompanying these outcomes that might further call attention to these outcomes and allow players to see through the LDW disguise. For these players who go from a fully effective disguise on LDWs in the positive game which they played first, to an “unmasking” of this disguise in the negative game which they played second, we see a dramatic reduction in win estimations.

The idea of the positive game when played first forming a template for celebratory sound frequency, and a seamless, effective disguise on LDWs that is then unmasked by the negative sounds in the second game may also account for player's significant affectual changes, but only when participants played the positive sounds game first. When participants played the negative sounds game first, positive affect, negative affect and flow did not differ between games. It is possible that the negative-sounds-first group are learning about how the game works, and do not experience the negative sounds as unduly negative since they have no positive game to compare it to. This would differ from those that are exposed to the positive game first. These participants are likely taken in by the LDW disguise in the positive sound game, and form a template as to how often they should hear positive sounds during game play. The high frequency of winning sounds, and the subsequent feeling that they are winning quite often would induce flow and positive affect, and likely reduce negative affect. Then, they play the negative sound game where positive sounds are fewer and further between. In this negative sound game, participants may notice the incongruity between negative sounds and positive animations in LDW outcomes. This may break flow and draw attention to these outcomes, resulting in participants looking more closely, and realizing that they are actually losing money on LDWs. This realization would result in a reduction of flow and positive affect, and an increase in negative affect. Importantly, these reductions in positive affect and flow cannot be due to habituation or fatigue, since these reductions were not observed in the negative sound first group.

In sum, this set of studies replicates past research demonstrating that participants respond to LDWs more similarly to wins when they are paired with positive sound, as they are in commercially available slot machines (Dixon et al. 2014a, 2015). More importantly, this set of studies provides evidence that pairing negative sounds with LDWs effectively lifts the disguise of these outcomes, as is seen through the PRP difference score analyses and reduced win estimations. In Experiment 1, we were able to show that by pairing negative sounds with losses and LDWs, participants were able to use sound as a single order categorization cue, since they were responded to in the same way as losses. Since the manipulation in Experiment 1 required adding negative sounds to 86.5% of spins, Experiment 2 investigated whether we could lift the disguise of LDWs by using a much more subtle sound manipulation, by only adding negative sound to LDW outcomes, or 19.5% of spins. This manipulation was still effective at lifting the LDW disguise—participants responded to these outcomes in a less win-like fashion, as can be seen through the PRP difference score analyses and reduced win estimates.

### Limitations

While the present studies enhance our knowledge around how sound impacts players’ responses to LDW outcomes, there are key limitations to note and additional questions to be answered. One limitation is the size of our sample. While this sample was still large enough to detect significant and meaningful effects, the present results should be replicated with a larger sample of participants. Further, the participants in our studies were relatively inexperienced slot machine players, and this may have impacted our results. For example, we were only able to show clear changes in affect and win estimations in order 1, where participants played the positive game first. It is possible that the lack of change in affect and win estimations for order 2 participants was due to unfamiliarity with slot machines in general. We would hypothesize that in a group of experienced gamblers, negative sounds may have a more substantial impact, even when played first. It is also important to note that our sample was entirely made up of undergraduate psychology students. Therefore, we consider these results a preliminary step towards further investigations which may utilize samples of more experienced gamblers recruited from community settings, or perhaps individuals experiencing varying levels of gambling harm.

Another important limitation to both studies is the fact that participants were not risking their own money to play, as would be the case with real slot machines. Thus, in our studies, participants were only playing with the potential to gain money and not playing with the potential to lose money. While we attempted to mitigate the impact of this limitation by including the opportunity to gain money based on their final balance on the slot machine, this limitation may still impede the ecological validity of our studies since participants were not playing under the exact same conditions as they would in a real-life situation.

On a related note, while we sought to create a high level of ecological validity by using real slot machine encasings, it is also important to acknowledge that the environmental conditions under which participants were playing were vastly different from the conditions under which they would play in an actual casino. For example, in a casino environment, gamblers are surrounded by many other people who are also gambling, which may impact their experience of outcome-specific sounds. Future studies may benefit from testing the impact of negative sounds following LDWs on gambler affect and behaviour within an even more realistic casino environment.

### Future Directions

Future studies could investigate how the presence of eye-catching animations following LDWs may influence participants. Due to technological limitations of our simulator, the negative sound conditions in our studies were still paired with the positive dancing animations. While the negative sounds were overwhelmingly negative, it is possible that some participants used these animations as a cue to determine whether they had encountered a loss or a win, thus diminishing the impact of our sound manipulation. Perhaps we did not see an effect on win estimations in the negative sound condition (when played first) because participants were using the animations instead of sound valence as a cue to determine whether they won or lost.

Additionally, while this study provides evidence that negative sound impacts players’ positive and negative affect in comparison to a positive sound game, it remains unknown whether this change in affect differs from baseline. Future studies should prioritize incorporating baseline affect measurements to assess how various features of slot machine games, such as positive or negative sounds, may influence slot machine gamblers. Additionally, future studies should address whether a negative sound manipulation might impact how long gamblers will voluntarily play a machine (e.g., behavioural persistence; Graydon et al. 2018), or whether the addition of negative sounds may influence a participants’ categorization of LDWs in a spin categorization task.

## Conclusion

In sum, this set of studies adds to our scientific knowledge of the influence of sound in multiline slot machines, particularly how sound influences the way in which participants behaviourally and subjectively respond to LDWs. These are the first experiments that have investigated how sound influences PRPs. Both studies found that by pairing LDW outcomes with a negative sound, PRPs are rendered more loss-like and less win-like. Both studies also found that the addition of negative sound results in higher fidelity win estimates. Moreover, it is not necessary to pair both regular losses and LDWs with negative sounds to unmask the LDWs. In Experiment 2, we found effects on PRPs and win estimate results by only pairing LDWs (19.5% of spins) with the negative sound. These studies demonstrate that negative sound is a viable way of unmasking the disguise of LDWs, resulting in participants responding to them as the losses they are rather than the wins they seem.
